# Ambulance clinicians’ responsibility when encountering patients in a suicidal process

**DOI:** 10.1177/09697330221149102

**Published:** 2023-04-07

**Authors:** Staffan Hammarbäck, Mats Holmberg, Lena Wiklund Gustin, Anders Bremer

**Affiliations:** Faculty of Health and Life Sciences, 427813 Linnaeus University, Växjö, Sweden; Centre of Interprofessional Collaboration within Emergency care (CICE), 427813 Linnaeus University, Växjö, Sweden; Department of Ambulance Service, 6140 Region Sörmland, Katrineholm, Sweden; Centre for Clinical Research Sörmland, Uppsala University, Eskilstuna, Sweden; Faculty of Health and Life Sciences, 427813 Linnaeus University, Växjö, Sweden; Centre of Interprofessional Collaboration within Emergency care (CICE), 427813 Linnaeus University, Växjö, Sweden; Department of Ambulance Service, 6140 Region Sörmland, Katrineholm, Sweden; Centre for Clinical Research Sörmland, Uppsala University, Eskilstuna, Sweden; School of Health, Care and Social Welfare, Mälardalen University, Esilstuna/Västerås, Sweden; School of Health, Care and Social Welfare, 8177 Mälardalen University, Västerås, Sweden; Department of Health and Care Sciences, UiT/The Arctic University of Norway, Tromsö, Norway; Department of Health and Caring Sciences, 427813 Linnaeus University, Växjö, Sweden; Centre of Interprofessional Collaboration within Emergency Care, 427813 Linnaeus University, Växjö, Sweden

**Keywords:** Ambulance clinicians, emergency medical services, ethical responsibility, phenomenography, suicidal ideation

## Abstract

**Background:**

Even though the traditional focus in emergency care is on life-threatening medical crisis, ambulance clinicians frequently encounter patients with mental illness, including suicidal ideation. A suicide is preceded by a complex process where most of the suicidal ideation is invisible to others. However, as most patients seek healthcare in the year before suicide, ambulance clinicians could have an important part to play in preventing suicide, as they encounter patients in different phases of the suicidal process.

**Aim:**

The aim of this study was to describe ambulance clinicians’ conceptions of responsibility when encountering patients in a suicidal process.

**Research design:**

A qualitative inductive design using a phenomenographic approach was used.

**Participants and research context:**

Twenty-seven ambulance clinicians from two regions in southern Sweden were interviewed.

**Ethical considerations:**

The study was approved by the Swedish Ethical Review Authority.

**Findings:**

Three categories of descriptions captured a movement from responding to a biological being to responding to a social being. Conventional responsibility was perceived as a primary responsibility for emergency care. In conditional responsibility, the patient’s mental illness was given only limited importance and only if certain conditions were met. Ethical responsibility was perceived to have its primary focus on the encounter with the patient and listening to the patient’s life story.

**Conclusions:**

An ethical responsibility is favourable regarding suicide prevention in ambulance care, and competence development in mental illness and conversation skills could enable ambulance clinicians to have conversations with patients about suicidal ideation.

## Introduction

Depending on the organization of the ambulance service, the professional affiliation of those working as ambulance clinicians (AC) differs between countries. Those who usually work within the ambulance service are medical technicians, paramedics, doctors and registered nurses.^1,2^ In ambulance care, medical needs are commonly prioritized, yet ACs frequently encounter patients with mental illness, including suicidal ideation. As most patients seek healthcare in the year before suicide, ACs could have an important part to play in suicide prevention if they have the right conditions and regard this as a task within the scope of their professional practice. However, ACs have described lack of competence regarding mental illness, and little is known on how they conceptualize their responsibility when encountering patients in a suicidal process.

### Background

Globally, over 700,000 persons die from suicide every year.^3^ In Sweden, the total number of suicides per year is estimated to be close to 1,600 persons, which means an incidence of 15 suicides per 100,000 inhabitants.^4^ A suicide is preceded by a suicidal process in which most of the suicidal ideation is invisible to others.^5^ In this study, suicidal ideation is understood in line with the definition in Diagnostic and Statistical Manual of mental disorders^6^ as thoughts about self-harm, with deliberate consideration or planning of possible techniques of causing one’s own death. The suicidal process might also involve a suicidal behaviour, which is any action that could cause the person’s own death. Commonly, a suicidal process has a duration of months or years. Still, most people with suicidal ideation do not attempt suicide.

There are several risk factors for suicide, including biological, psychological, social and environmental factors.^7^ Even though the risk for suicide is higher with conditions such as severe depression and a history of self-harm, far from all who die from suicide have a psychiatric diagnosis.^3^ Suicidal ideations can also be associated with an experience of one’s life situation as being unendurable without the person being mentally ill. The concept of mental illness will be used to include the width of both psychiatric and existential mental ill-health conditions.

An impending suicide is often communicated, although not necessarily verbally.^8^ Thus, it is possible and desirable for clinicians to develop an understanding of the patient’s inner state to discover non-verbal signs of impending suicide.^9^ Patients have described that communication about suicidal ideation can be a form of relief, while not being able to talk about it can increase the agony.^10^ Regaining hope is associated with the experience of being met by clinicians who truly listen. As most patients encounter healthcare in the year before suicide, it is important that clinicians are observant of suicidal ideation.^11^ This becomes even more important when considering that ambulance services could be the only contact patients have with the healthcare system before taking their lives.^12^

Traditionally in emergency care, focus is on treating life-threatening medical crisis which might impede the ACs from assessing the patient’s suicidal ideation.^13^ When patients in a suicidal process encounter ambulance care, they encounter ACs that value abilities and professional ideals such as swiftness, technical skills, self-confidence and determination.^14^ ACs frequently encounter patients with mental illness, including suicidal ideation, but they experience lack of competence and system failure for these patients.^15^ They strive to do both what is best for the patient, but also to do what is expected from them as professionals. Patients with suicidal behaviour can challenge the way ACs perceive their role. In emergency care there seems to be a distinction of perceived legitimate and illegitimate calls with medical needs tending to be prioritized.^16^ The Swedish description of competence for prehospital emergency nurses^17^ states that ACs need an ethical approach that prerequisites a personal responsibility and ACs’ care is motivated by the patient’s physical, psychological, social and existential needs. Perceptions of responsibility in ACs are grounded in medical science but include aspects of nursing care and collaboration with other healthcare professionals as well.^18^

Suicide prevention in itself, and caring for suicidal patients is clinically and ethically challenging, as the conditions are complex and calls for a range of decisions.^19^ Ethical challenges include clinical, relational and organizational factors. ACs could have an important part to play in preventing suicide, as they encounter patients in different phases of the suicidal process. The first encounter with the healthcare system after an attempted suicide is most likely of great importance to the patient, and can sometimes open a window in which it is possible to talk openly about the situation.^20^ However, encountering patients is a suicidal process could be challenging, and there is to our best knowledge a gap in the current research as little is known about how ACs conceptualize their responsibility in these situations. Therefore, the aim of this study was to describe ACs’ conceptions of responsibility when encountering patients in a suicidal process.

### Research design

This study has a qualitative inductive design using a phenomenographic approach.

## Methodology

Phenomenography is rooted in the science of pedagogy and makes an epistemological assumption that there are relatively limited ways of conceptualizing qualitatively different aspects of reality or phenomena.^21^ Marton distinguishes between first order perspective and second order perspective where the former perspective focuses on the phenomenon itself, while the latter perspective has its focus on how the phenomenon is conceptualized. Taking on a phenomenographic approach, it is only the second order perspective that is of interest, leaving out what the phenomenon is by itself. The research approach aims at finding and systemizing patterns in peoples’ ways of thinking without classifying or making fair or unfair judgement of the participants. The most central concept of the research approach is conception, and this is understood as a person’s way of understanding and perceiving a phenomenon. A conception is a taken for granted way of understanding a phenomenon, and it is the non-reflected foundation of actions and reasoning.

### Participants and research context

As phenomenography aims at describing the variations of conceptions, a purposeful sampling was chosen to reach variation in characteristics, including varying educational levels, among the participants.^22^ The ambulance care organisation in Sweden is regulated by the Swedish healthcare act, and each of the 21 regions are responsible for providing ambulance care to its residents. The participants were ACs and worked in two regions including four districts and a total of six ambulance unit stations in southern Sweden. The stations were equipped with between one and four ambulances and were placed in both smaller and larger cities, varying between 3,000 residents and 110,000 residents. The stations covered both urban and rural areas. No assistance from prehospital psychiatric resources was available within the ambulance organizations. The first author provided information about the study to the staff at the ambulance stations. Contact with possible participants was facilitated by the unit managers who were asked to identify staff members with a variety of competence, age, gender and work experience. Altogether, the unit managers found 27 persons who were interested in participating in the study and there were no dropouts Table 1.

### Data collection

Data were collected between September and December 2019. As the method of data collection, the first author performed individual semi-structured interviews with open-ended questions. A pilot interview, not included in the study, was conducted to test the interview guide. The interviews included three areas of questions: *assignments*, *conversations,* and *responsibility*. The interviews started with the question: *‘What are your thoughts on encountering patients in a suicidal process?’* Follow-up questions were asked based on the participants’ unique answers. The participants themselves chose the location of the face to face interview, either at the participant’s workplace (*n* = 22) or at the participant’s home (*n* = 5). The interviews lasted between 14 and 62 min (mean 30 min) and were audio recorded and transcribed verbatim.

**Table 1.** Characteristics of participants (*n* = 27).


Gender, *n* (%)	
Male	15 (56)
Female	12 (44)
Age groups (years), *n* (%) [range]	[29–59]
*≤*30	1 (4)
31–40	8 (30)
41–50	9 (33)
≥51	9 (33)
Ambulance work experience, *n* (%) [range]	[1–32]
*≤*5	8 (30)
6–10	3 (11)
11–15	7 (26)
16–20	5 (19)
≥21	4 (15)
Experience from psychiatric care, *n* (%)	9 (33)
Education and profession, *n* (%)	
EMT^a^	4 (15)
RN^b^ without specialist education	6 (22)
RN with one type of specialist nurse education	15 (56)
RN with two or more types of specialist nurse education	2 (7)

^a^Emergency Medical Technician.

^b^Registered Nurse.

### Data analysis

The aim of phenomenographic analysis is to discover patterns in the participants’ way of thinking and relating to a phenomenon and not to directly describe their separate experiences of the phenomenon.^22^ The analysis was grounded in the steps described by Dahlgren and Fallsberg.^23^ The first author conducted the analysis with reoccurring discussion and supervision from the other authors. First, the transcripts were read through several times, and spontaneous thoughts of the separate interviews were written down. In the next step, all the conceptions that were found were marked, given a code and copied to an Excel document. Thereafter, the section that included a conception was labelled which facilitated the grouping of similar conceptions. The different groups were then compared both internally and externally, and in that process, three different categories of descriptions emerged. Three out of four authors have experience from ambulance care which could impact on confirmability.^24^ During the categorizing the authors went back to the original transcripts to ensure that the findings were derived from data and not constructed from preunderstandings. Still, it may not be completely possible to put aside years of experience, but at the same time, being familiar with the context could be considered a strength. The categories were discussed within the research group and no overlap was found. Thereafter, the structural relation between the categories was defined. This comparison between the categories of descriptions constitute the outcome space in which the relation between the categories is made clear and visible. No statements were made on whether the conceptions are to be considered true or false. Examples from the analysis process are presented in Table 2.

**Figure 1.**
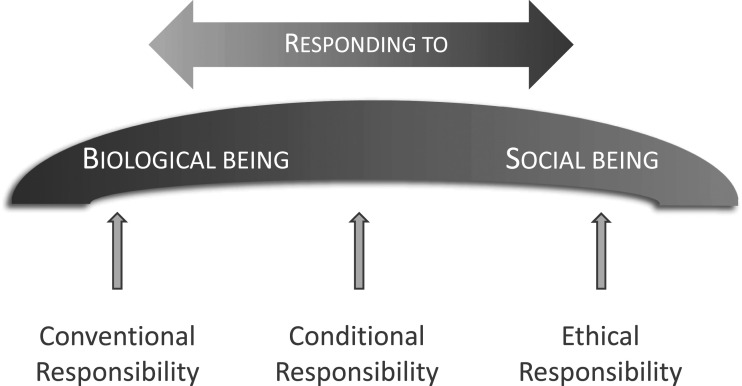
Outcome space.

**Table 2.** Examples from the analysis process.

Conception	Code	Category
A purely informative conversation. First and foremost to gather much information, to gather data to me and the medical record and to my handover report. (AC22)	Conversation to gather information	Conventional responsibility
Then my responsibility is to actually pass on, when I get to the emergency room, that I think there is a lot of mental illness, there is a lot of loneliness problems, so when the doctor has checked things, you should get a referral to psychiatry or get help in some way, that is my responsibility. (AC8)	Responsibility to report suspicions	Conditional responsibility
So if you have a gut feeling, then I think that I have a responsibility, then I have a huge responsibility to ask that question. (AC6)	Asking about suicidal ideation is intuitive	Ethical responsibility

### Ethical considerations

The study was approved by the Swedish Ethical Review Authority (No 2019-03,774) and was performed in accordance with the Declaration of Helsinki.^25^ Unit managers facilitated the contact with participants; however, the question of participation was asked by the first author. Before conducting the interviews, the participants were given both verbal and written information about the study and were told that their participation was voluntary. They were given information about their rights to withdraw their participation at any time without stating their reasons. There were no withdrawn participations. During the analysis and forward, the participants’ names were anonymized and replaced with a code. The code key was only accessible to the first author. Before starting the interviews, a written consent was signed by the participants.

## Findings

Three categories of descriptions were found. *Conventional responsibility*, where responsibility is mainly conceptualized as linked to the ACs’ role as a provider of emergency care. ACs are not perceived as having the right competence to help patients with their suicidal ideation. *Conditional responsibility* includes a mandatory responsibility of physical assessment and treatment and when completed there is an optional aspect of including mental illness in care. Embracing conversations about suicidal ideation is linked to conditions such as the ACs’ own perceived competence. *Ethical responsibility* is conceptualized as related to the ACs as primarily presenting to the patients as fellow human beings. Establishing a relationship with the patient is considered as a main purpose and there is a responsibility to create time and space for the patient’s story to be shared. The categories’ relation to each other is elucidated in the outcome space (Figure 1) and their internal characteristics are described separately.

### Categories of descriptions

#### Conventional responsibility

Conventional responsibility derives from formal guidelines and algorithms and includes the physical aspects linked to suicidal behaviour. This means having responsibility for emergency care at the scene and most prominently responding to the patient’s medical condition.

*“That is not the reason I am there, to take care of someone in any deeper way, so to say, mentally (…) I am there for emergency medicine of course.”* (AC7)

Easing the patient’s suffering is understood as requiring a certain competence in conversation skills. This competence, and thus responsibility, is perceived as being found within psychiatric care and among psychiatric clinicians. The AC’s responsibility is conceptualized as being related to the level of emergency care that the patient needs.

*“I guess it is no secret that most of us see ourselves as some somatic function, so then you sort of want some kind of somatic incident or trauma linked to the suicide attempt for it to sort of become a legitimate ambulance service patient.”* (AC24)

Asking the patient for suicidal ideation is motivated if suicidal expression is mentioned in the information from the medical dispatch centre. The question aims to determine whether there is true intention to die, or an expression of mental illness. There is no responsibility to ask for suicidal ideation when causes of contact are due to medical conditions. The primary goal of the conversation is to gather information about the patient. Since suicidal behaviour is understood as making patients erratic and possibly change their stories, there is a responsibility to document suicidal ideation when it has been revealed.

There are a group of patients who are conceptualized as only crying for help without having genuine suicidal intentions. Responsibility when encountering these patients is understood as preventing them from using ambulance care due to mental illness.

*“It feels like you cannot give them too much attention because then they find a channel where there is someone who cares and then they are going to misuse that channel and call for an ambulance every day or every week.”* (AC10)

Responsibility is understood as emanated from the professional role to provide emergency care and the ACs avoid getting personally involved.

#### Conditional responsibility

If certain conditions are met, the ACs can to a limited extent include the responsibility to care for the patient’s mental illness while being in a suicidal process. What happens in the encounter between the AC and the patient is understood as having an effect on the patient’s onward care and thus a responsibility arises to represent the healthcare system. Having template questions concerning suicidal ideation is understood as clarifying responsibility. Primarily, responsibility is held over the physical aspects but when these have been attended to, focus switches to mental illness. The responsibility of care is understood as instilling hope and trust in the healthcare system. However, the responsibility to represent the healthcare system is only upheld if there is a conception that the rest of the system takes proper responsibility of their parts in the patient’s care. The risk of reduction in the conceptualized responsibility is especially prominent when the psychiatric care appears to have failed, for instance when the patient expresses that they have not been helped by psychiatric care, or are recurrently in need of ambulance care due to suicidal behaviour.

*“They make another attempt because they did not get help. Then it gets very hard for me to once again refer to psychiatry.”* (AC27)

Responsibility for having a conversation, as part of caring for the patient, is also conceptualized as conditional if, for instance, the environment prerequisites conversations. The responsibility is in relation to the ACs’ own perceived competence, resulting in greater responsibility with higher perceived competence. There is a similar understanding concerning time, where less time with the patient means less responsibility for conversations and questions about suicidal ideation. Conversations are therefore not conceptualized as a mandatory responsibility. However, the more likely the patient is perceived as being in a suicidal process, the greater the responsibility to ask about it. If the question is asked, then a responsibility arises to respond and handle the answer in a professional way. Since the responsibility is not perceived as mandatory, there are situations where the ACs can opt out from asking about suicidal ideation.

*“There are likely occasions where I have felt that no, I am not going to ask that question because I do not know what to do with the answer, or my work effort will be so increased.”* (AC18)

If the question is not asked, there is still a responsibility to report any suspicions of suicidal ideation.

#### Ethical responsibility

Ethical responsibility arises from the relationship with the patient and recognising the patient´s suffering calls for a response to help as a fellow human being. Responsibility is understood as an ethical demand to not abandon the patient but to confirm and to convey hope. It is by establishing a relationship that all aspects of the patient can be embraced. In compassion, the ACs strive to earn the patient´s trust and they allow themselves to be vulnerable in the patient relationship.

*“You sort of get, you get vulnerable yourself somehow because I am going in with my personal… it is not just, it is not just my professional role but in some way, I reveal myself.”* (AC15)

The patient is understood as having a life story to tell and the ACs have a responsibility to ensure the patient feels secure enough to share this story and to show the patient that the ACs have time to listen. The conversation is understood as pivotal in the patient relationship and emanates from the patient’s story. An established relationship may ease a conversation about suicidal ideation. At the same time the conversation about suicidal ideation is perceived as deepening the relationship. The conversation is understood as having the possibility to ease the patient’s suffering. Thus, the ethical responsibility includes listening attentively and being completely present. Listening to the patient’s life story is perceived as helping the patient get perspective of the situation and identify needs and possibilities.

*“They get to put their feelings into words, I think sometimes there is a difference when you feel something inside or when you say it out loud. I think it is very good if they get to put their feelings into words, why this has happened or why they feel like this and get to share that with someone.”* (AC5)

The question about suicidal ideation is to a high degree controlled by an intuitive process and the responsibility to ask exists even when encountering a patient who shows no signs of suicidal behaviour. Work experience, reflection, knowledge and training in mental health and suicidology are perceived as decreasing the sense of vulnerability, and refining the ability to sense suicidal ideation and asking the patient is motivated as it can potentially save a life.

*“What I learned when I did the training, that you should actually dare to ask about it, you should dare to talk about it.”* (AC13)

### Outcome space

Responsibility is understood as responding to something. When comparing the three categories of descriptions, they have different qualitative conceptions of responsibility, and move from *responding to a biological being* to *responding to a social being* (Figure 1). Conventional responsibility is located at responding to a biological being, where focus lies on the physical aspects of the patient. At the other end of the scale, responding to a social being, you can find ethical responsibility. Here responsibility derives from the relationship with the patient, and it is the patient’s need of care that awakens the ACs’ responsibility. The conditional responsibility is located in between the other categories and primarily responds to a biological being but can under certain conditions include the patient as a social being. ACs describe a movement across the spectrum of responsibilities, making them prominent in different situations. Responding to a social being is considered to require a relationship and being able to have a conversation is seen as a key aspect when establishing the relationship. If the ACs cannot utilise this capability, for example because of tiredness or language deficits, responsibility tends to move from a social being to a biological being.

*“You are quite exhausted (…) you cannot really take hold of certain things, but you distance yourself.”* (AC2)

At the same time there are conditions which facilitate the relationship and the ability to have conversations. Examples of such conditions are working with a supportive colleague and training in mental health and conversation skills. These conditions move the responsibility to responding to a social being.

## Discussion

The present result shows variety in how responsibility is conceptualized and moves between responding to a biological being and responding to a social being. When responding to a biological being, the focus of the assignment is to provide emergency care. This is in line with Bolster et al.,^26^ who point out that in emergency care, patients with suicidal problems are not considered as seriously ill. Bolster et al. stress that this view of suicidal ideation in emergency care could be lethal. A troubling fact is that previous research shows that patients with suicidal ideation are more likely to encounter emergency care than mental care.^27^

In the movement between responding to a biological being and responding to a social being, the present result implies a limit to the extent the patient’s mental illness was included in the care. This accords with previous observations by Todorova et al.,^28^ who describe how ACs concentrated their assessment and treatment on medical disorders while contemporary mental illness was considered secondary and a perceived lack of knowledge in mental illness called for competence development. Not having the right competence when encountering patients with suicidal ideation has been described by Rees et al.,^15^ and the present result shows that knowledge and training were perceived as facilitating engaging in relationships and asking about suicidal ideation. Such engagement is not only a matter of making suicide assessment. Following Vatne and Nåden^29^ encounters like the ones associated with an ethical responsibility can also contribute to patients’ experiences of hope. In conformity with Holmberg et al.,^30^ ACs adjust their care according to their colleague and this could either facilitate or inhibit engaging in a patient relationship. The present result indicates a conception of engaging in a patient relationship as requiring energy and empathy. Therefore, ethical responsibility is harder when ACs perceive tiredness or hopelessness. ACs are commonly exposed to patient pain and suffering, and have described ethical issues regarding patient relationships when they are not able to provide the help they wish they could.^31^ Sabo^32^ describes such circumstances as possibly causing compassion fatigue. In the present result ACs describe frustration and distancing when they conceptualize that they cannot help the patients, as they themselves lack the right competence and are not either able to refer to adequate competence.

The ethics in ethical responsibility emerges from the patient relationship and the patient´s suffering, as it calls for a response from the ACs as human beings. This is in line with Gallagher^33^ who describes the ethics of compassion as an important addition to the traditional principles of ethics in healthcare. Relation ethics is also described by Birrell and Bruns^34^ as something beyond ethical codes, and they argue that ethics is already embedded in the encounter. Relational ethics requires compassion and the ability to listen, not only to understand but to move beyond the already known into uncertainty. In ethical responsibility, complete presence and attentive listening to the patient’s life story is perceived as imperative as it might reveal events that indicate that the patient is at higher risk of suicide, for example due to employment loss or other stressful life-events.^35^ ACs also describe the responsibility of listening as helping the patient put their feelings into words. This is in line with Holopainen et al.,^36^ and their description of the caring encounter as including an ethical responsibility to be present and open for the patient’s narrative of suffering. Ganzini et al.,^37^ found that veterans were more likely to disclose suicidal ideation when they experienced that the clinicians expressed genuine concern, personal interest and had a caring attitude. Richards et al.,^38^ describe further support for the importance of patients experiencing a trusted relationship, caring attitude and someone truly listening if they are to disclose suicidal ideation.

The present result shows that ACs feel vulnerable as they engage in a patient relationship with a patient in a suicidal process. This is in line with Angel and Vatne^39^ who describe how in the clinician-patient relationship, traditional focus has been on the vulnerability of the patient while the clinician is expected to be the strong part. However, clinicians may experience vulnerability as well, especially when engaging in a patient relationship and risking the experience of not being able to manage the situation. Still, vulnerability is not equal to being weak but could instead be understood as a way of being compassionate and able to go with the patient to where it hurts.^40^ Emotions can thus be resources when taking an ethical responsibility in the patient relationship. When clinicians allow themselves to be touched by the patient as another human being, and to become emotionally involved in the patient, an experience of ethical responsibility can arise and an appropriate response is required.^41^ When responsibility is understood as responding to a biological being, ACs protect their own vulnerability as focus is on emergency care, while the patient relationship is omitted. Bolster et al.,^26^ point out that avoiding asking about suicidal ideation could be connected to having a fear of not being able to help or a perception that saying the wrong thing could cause the patient further harm.

Even though conventional and conditional responsibility responds primarily to the patient as a biological being, they are not to be understood as unethical. ACs face different kinds of ethical conflicts while striving to provide care for patients in need, including deciding what is in the patient’s best interest.^42^ Avoiding conversations about suicidal ideation can be related to the ethical principle of non-maleficence. When ACs perceive that they do not have the right competence for having conversations about suicidal ideation, it is understood that such conversations could cause the patient harm. Therefore, these conversations should be held with clinicians with the appropriate competence. This is line with the ICN Code of Ethics for Nurses^43^ and the statement that nurses should practice within the limits of their individual competence. However, the code also states that nurses are patient advocates, which is more in line with the category of ethical responsibility.

Suicidal ideation and ambivalence about living as well as suicidal behaviour are risk factors of suicide and early recognition is therefore utterly important.^44^ ACs have a unique opportunity to investigate suicidal ideation as they are often alone with just one patient. Furthermore, they frequently encounter patients with risk factors of suicide such as old age and comorbidity.^45^ However, being strongly influenced by the understanding of the patient as a biological being, questions about suicidal ideation are usually not asked. Enhancing ethical responsibility when encountering patients in a suicidal process could be beneficial, not only to prevent suicide, but to instil hope. When understanding responsibility as responding to the patient as a social being rather than merely a biological being, ACs could through attentive listening, help the patient to ease their burden and suffering.

## Limitations

A considered limitation could be that no participants were recruited from major cities since these ambulance services tend to have a higher workload with less time for reflection and recovery. It is also possible that working in organizations with assistance from prehospital psychiatric resources could affect conceptions of responsibility. Not having participants from such organizations could be a limitation.

Concerning dependability, we find that similar conceptions would be found if the study were to be replicated with similar participants and in similar contexts.^24^ However, we recognize the possibility of different categorization and naming. Participants were not given the opportunity to give feedback to the findings, which can be considered a weakness in terms of the confirmability of the results. However, getting feedback from participants on the findings might reveal more of their individual opinions and attitudes, while a phenomenographic approach aims to describe participants’ conceptions of a phenomenon.

Concerning the traditional medical focus in ambulance care, we found it plausible that similar findings would occur in other contexts with the same traditions, for instance in other emergency care contexts, which can be assumed to strengthen the transferability of the results.

## Implications

• Promoting ethical responsibility could prerequisite suicide prevention in ambulance care.
• ACs need competence development in mental illness and conversation skills to facilitate ethical responsibility and conversations about suicidal ideation.
• ACs need clarified responsibility together with interprofessional collaboration within a well-functioning healthcare system when they encounter patients in a suicidal process.
• ACs need organizational and collegial support including time for reflection to ease their vulnerability when encountering patients in suicidal processes.

## Conclusion

There is variety in how ACs conceptualize their competence to have conversations about suicidal ideation. ACs express either a lack of competence or that competence development has facilitated these conversations. A prerequisite for having conversations is a perceived competence to meet and handle what the patient might say. However, the perceived competence is related to conditions in the ACs’ work situation, such as tiredness, support of colleagues and a feeling of being part of a well-functioning system. Conceptualizing responsibility as an ethical demand and emerging from the patient´s suffering facilitated engaging in a patient relationship. This could be vital as it is in the patient relationship that suicidal ideation could be disclosed and understood.

Further research is needed regarding ACs’ motivation and attitudes to engage in relationships and conversations with patients in a suicidal process. There is also a lack of research concerning experiences of the encounter between ACs and patients in a suicidal process, from the perspective of the latter.
